# Protocol for multiplexed detection of antibody binding to cell-surface-expressed hemagglutinin by flow cytometry

**DOI:** 10.1016/j.xpro.2026.104614

**Published:** 2026-06-04

**Authors:** Leon Ullrich, Katharina Daniel, Anna-Lena Schumacher, Maximilian Germer, Florian Klein, Christoph Kreer

**Affiliations:** 1 Laboratory of Experimental Immunology, Institute of Virology, Faculty of Medicine and University Hospital Cologne, University of Cologne Cologne 50931, Germany; 2 FACS & Imaging Core Facility, Max Planck Institute for Biology of Ageing Cologne 50931, Germany; 3 German Center for Infection Research (DZIF), Partner Site Bonn-Cologne, Cologne, Germany; 4 Laboratory for Immunodynamics and Molecular Evolution, Institute of Virology, Faculty of Medicine and University Hospital Cologne, University of Cologne Cologne 50931, Germany

**Keywords:** Cell-based Assays, Flow Cytometry, High Throughput Screening, Immunology, Antibody

## Abstract

Measuring antibody binding titers to viral proteins is essential to assess population immunity and monitor viral escape. We present a multiplexable flow cytometry protocol to quantify antibody binding to native-like, cell-surface-expressed antigens and demonstrate the method using influenza A virus hemagglutinin. We describe steps for the transfection of human suspension cells, cell staining and fixation, data acquisition, and analysis. In a single run, up to 500 samples can be measured simultaneously against three antigens per well within three days.

For complete details on the use and execution of this protocol, please refer to Daniel et al.^1^

## Highlights

• Steps for HEK293-6E cell transfection with antigen-fluorescent protein plasmids
• Detailed instructions on binding assay setup with an example plate layout
• Steps to prepare gating/compensation controls and acquire samples on a flow cytometer
• Guidance on gating strategy and curve fitting to derive EC_50_/ED_50_ values

## Abstract (editor highlights)

Publisher’s note: Undertaking any experimental protocol requires adherence to local institutional guidelines for laboratory safety and ethics.

## Graphical abstract

**Figure:**
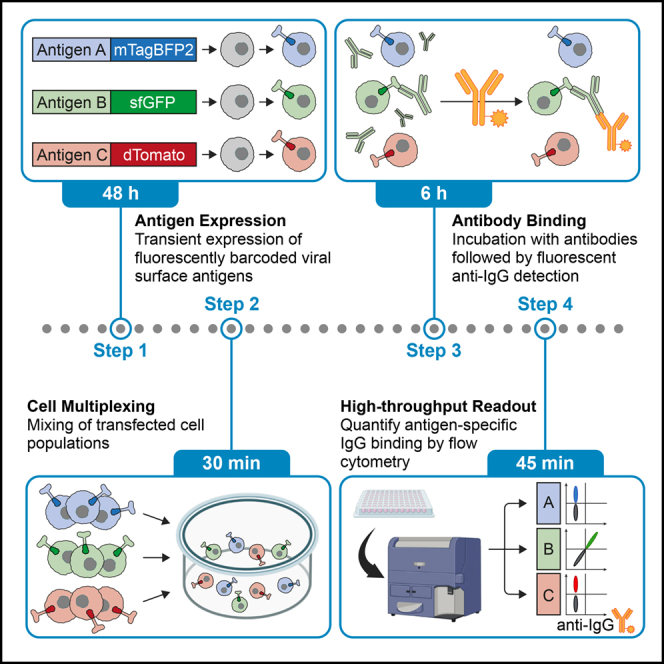

## Before you begin

This assay is based on the flow cytometric detection of antibody binding to viral antigens in a native-like context on the surface of transfected mammalian cells. Antigen-expressing cells are identified by a fluorescent reporter protein fused to the intracellular C terminus of the antigen. Different antigen variants can be analyzed in parallel by separately transfecting cells with distinct antigen-fluorescent protein combinations. The linked fluorescent proteins function as barcodes, allowing mixed cell populations to be deconvoluted during flow cytometric analysis. Binding of mono- or polyclonal antibodies to surface-expressed antigens is detected using a fluorophore-conjugated secondary anti-human IgG antibody. Titration of monoclonal antibodies or serum/plasma samples can be used to determine half-maximal effective concentrations or dilutions.

The protocol below describes the specific steps for testing the binding of monoclonal antibodies or human sera/plasma samples against the hemagglutinin (HA) of influenza A virus. The workflow is optimized for transfection of HEK293-6E suspension cells, which provide high transfection efficiency and support robust assay performance. However, the protocol can easily be adapted to other antigens or cell types.

### Innovation

The presented assay overcomes key limitations of standard ELISA-based techniques by avoiding protein purification and enabling multiplexed detection of viral target proteins on fluorescently barcoded cells. A modular plasmid design allows rapid exchange of both viral target proteins and fluorescent proteins, facilitating adaptation to specific experimental requirements and available flow cytometer platforms. Multi-well plate-based workflows enable convenient scaling and allow up to 500 individual tests to be performed within three days. In addition, fixation provides enhanced biosafety and permits acquisition for up to seven days after staining. In this protocol, hemagglutinin (HA) is used as a model target protein for the detection of human polyclonal and monoclonal antibodies. However, the assay can be readily adapted to other viral proteins and species by exchanging the target construct and the corresponding detection antibody. While experimentally validated using three fluorescent proteins on a conventional filter-based flow cytometer, further multiplexing may be feasible on spectral flow cytometry platforms.

### Institutional permissions

This protocol involves the generation of genetically modified organisms (GMOs), specifically human cell lines expressing viral surface proteins fused to fluorescent reporter proteins. In our case, these GMOs are classified as biosafety level 1 (BSL-1) and require documentation in accordance with institutional biosafety regulations. Researchers should verify local biosafety requirements, as additional approvals may be necessary depending on the nature of the constructs and regional regulations. In the original study by Daniel et al. 2026,^1^ polyclonal human sera were assessed. These sera were obtained from the EIKIM study (DRKS00026266), approved by the Institutional Review Board of the University of Cologne (approval number #21-1468). All participants provided written informed consent prior to sample collection. No animal-derived sera were analyzed in this protocol. If animal samples are included, appropriate approval from the relevant veterinary or animal welfare authorities may be required in accordance with local regulations.

### Cell culture of HEK293-6E cells

**Timing: ∼2 weeks**

On the day of transfection, HEK293-6E cells are required at a density of 0.8 × 10^6^ cells/mL (see step-by-step method details). This section describes thawing and routine suspension culture of HEK293-6E cells in FreeStyle™ 293 Expression Medium using vented Erlenmeyer flasks and incubation at 37°C, 6% CO_2_, with shaking at 110 rpm. In our experience, cells become reliably transfectable after ∼2 weeks of recovery and should routinely achieve ≥60% transfection efficiency for robust assay performance.

1. Thaw one cryovial of HEK293-6E cells (1 × 10^7^ cells/mL) in a 37°C water bath until only a small ice crystal remains and immediately transfer the cell suspension into 45 mL pre-warmed culture medium.

**CRITICAL:** Store cryovials long-term in vapor-phase liquid nitrogen (≤−150°C).

2. Centrifuge the cells at 400 × *g* for 5 min at 20–25°C.
3. Discard the supernatant and resuspend the cell pellet in 10 mL pre-warmed culture medium.
4. Recover cells in suspension culture by incubating at 37°C, 6% CO_2_, with shaking at 110 rpm in a 125 mL vented culture flask.
5. During the first 1–2 weeks after thawing, monitor cell density daily and adjust to 0.3 × 10^6^ cells/mL using fresh medium.

***Note:*** Do not allow cells to exceed 0.5 × 10^6^ cells/mL during this recovery phase.

***Note:*** Growth is often slow during the first seven days after thawing.

6. Passage cells every other day by diluting cultures back to 0.3 × 10^6^ cells/mL (or 0.2 × 10^6^ cells/mL before extended incubation periods, e.g., weekends).

***Note:*** In our experience, cells can be used reliably for transfection after ≥3 passages, provided that test transfections yield ≥60% efficiency after 48 h. We typically observe a decline in transfection performance at higher passage numbers (∼45–50). However, if transfection efficiencies decrease before reaching this passage range, we recommend thawing a fresh vial.

**CRITICAL:** Avoid overgrowth. If cultures exceed 1.8 × 10^6^ cells/mL, transfection performance may drop substantially and may remain reduced even after re-adjusting cell density. Discard overgrown cultures and thaw a fresh vial if transfection efficiencies decline.

### Plasmid design and availability

**Timing: ∼1 week (if plasmids are obtained from Addgene); 1–3 weeks if constructs need to be synthesized or newly cloned**

**Figure 1:**
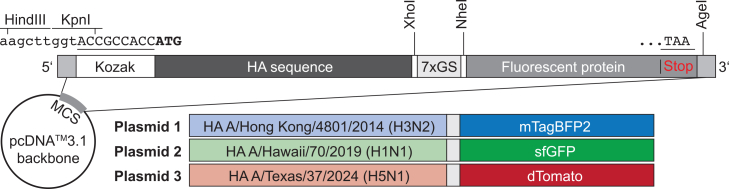
Modular design of HA–fluorescent protein expression constructs Schematic overview of the modular pcDNA™3.1-based expression vector used in this protocol. The construct contains a 5′ Kozak sequence followed by the hemagglutinin (HA) coding sequence including its endogenous signal peptide but lacking the endogenous stop codon. HA is fused in-frame via a 7×GS linker to a fluorescent protein module, which includes the stop codon (TAA). Restriction sites used for modular exchange of the antigen and fluorescent protein modules are indicated (HindIII/KpnI at the 5′ end, XhoI upstream of the linker, and NheI/AgeI flanking the fluorescent protein module). The three HA–fluorescent protein constructs used in Daniel et al. 2026^1^ are shown below (Plasmid 1: A/Hong Kong/4801/2014 (H3N2)–mTagBFP2; Plasmid 2: A/Hawaii/70/2019 (H1N1)–sfGFP; Plasmid 3: A/Texas/37/2024 (H5N1)–dTomato).

We developed a modular expression system based on the pcDNA™3.1 (Thermo Fisher Scientific) backbone to enable flexible exchange of viral antigens and fluorescent reporter proteins (Figure 1). The general construct architecture consists of a full Kozak consensus sequence upstream of the start codon, the viral antigen coding sequence including its endogenous signal peptide, removal of the endogenous stop codon, a C-terminal 7×GS linker, and an in-frame fluorescent protein containing its own stop codon.

Antigen sequences were codon-optimized for mammalian expression and synthesized without internal restriction sites to facilitate modular cloning (HindIII, KpnI, XhoI, NheI, and AgeI). Fluorescent protein sequences were similarly codon-optimized and restriction-site adapted.

In Daniel et al.,^1^ we generated three plasmids for the initial screening that encode three different HA variants, each fused to a distinct fluorescent protein (Plasmid 1: A/Hong Kong/4801/2014 (H3N2)–mTagBFP2; Plasmid 2: A/Hawaii/70/2019 (H1N1)–sfGFP; Plasmid 3: A/Texas/37/2024 (H5N1)–dTomato, Figure 1). These constructs have been deposited at Addgene under the names pcDNA3.1-HA-H3N2-mTagBFP2 (Plasmid 1), pcDNA3.1-HA-H1N1-sfGFP (Plasmid 2), and pcDNA3.1-HA-H5N1-dTomato (Plasmid 3) (accession numbers 253733–253735) to enable direct access and immediate use.

When generating alternative antigen–fluorescent protein combinations, ensure to remove the stop codon from the antigen sequence, include a Kozak sequence upstream of the start codon, maintain the correct reading frame, use compatible restriction sites for the modular cloning strategy, and verify the final construct by plasmid sequencing.

***Note:*** In addition to the deposited antigen–fluorescent protein plasmids, this protocol requires an unlabeled antigen-only plasmid (no fused fluorescent protein) for the compensation controls. In this protocol, this corresponds to an HA-only construct. This plasmid is not included in the Addgene deposit and must be provided by the investigator (e.g., by introducing a stop codon immediately downstream of the antigen coding sequence to remove the linker/fluorescent protein module).

## Key resources table

**Table:** 

REAGENT or RESOURCE	SOURCE	IDENTIFIER
**Antibodies**		

BD OptiBuild™ BV711 Mouse Anti-Human IgG, clone G18-145 (1:300 dilution)	BD Biosciences	Cat#740796; RRID: AB_2740459
CR6261	Throsby et al.^2^	N/A

**Chemicals, peptides, and recombinant proteins**		

4′,6-Diamidino-2-phenylindole (DAPI)	Miltenyi Biotec	Cat#130-111-570
Branched Polyethylenimine (PEI), 25 kDa	Sigma-Aldrich	Cat#408727 CAS: 9002-98-6
Cytofix™ fixation buffer	BD Biosciences	Cat# 554655
DPBS	Gibco	Cat#14190169
Ethylenediamine tetraacetic acid (EDTA)	Carl Roth	Cat#CN06.3
Fetal bovine serum (FBS)	Sigma-Aldrich	Cat#F9665
FreeStyle™ 293 Expression Medium	Thermo Fisher Scientific	Cat#12338026
PBS	Gibco	Cat#10010056
Penicillin-Streptomycin	Gibco	Cat#15070063
Zombie NIR™ (1:600 dilution)	BioLegend	Cat# 423106

**Experimental models: Cell lines**		

HEK293-6E cells	National Research Council of Canada	NRC file 11565

**Recombinant DNA**		

dTomato	Shaner et al.^3^	N/A
HA Sequence A/Hawaii/70/2019	GISAID	GISAID: EPI_ISL_400916
HA Sequence A/Hongkong/4801/2014	GISAID	GISAID: EPI_ISL_1655544
HA Sequence A/Texas/37/2024	GISAID	GISAID: EPI_ISL_19027114
mTagBFP2	Subach et al.^4^	N/A
pcDNA3.1-HA-H1N1-sfGFP	Daniel et al.^1^	Addgene #253733
pcDNA3.1-HA-H3N2-mTagBFP2	Daniel et al.^1^	Addgene #253734
pcDNA3.1-HA-H5N1-dTomato	Daniel et al.^1^	Addgene #253735
pcDNA™3.1/V5-His TOPO™ TA Expression Kit	Thermo Fisher Scientific	Cat#K480001
SuperfolderGFP	Pedelacq et al.^5^	N/A

**Software and algorithms**		

FACSDiva 9.0.1	https://www.bdbiosciences.com/en-us/products/software/instrument-software/bd-facsdiva-software	RRID: SCR_001456
FlowJo 10.10.0	https://www.flowjo.com	RRID: SCR_008520
Microsoft Excel for Mac (V16.78.3)	https://www.microsoft.com/de-de/microsoft-365/mac/microsoft-365-for-mac	N/A
Prism 10	https://www.graphpad.com	RRID: SCR_002798
SnapGene	https://www.snapgene.com	RRID: SCR_015052
VectorBuilder Codon Optimization Tool	https://en.vectorbuilder.com/tool/codon-optimization.html	N/A

**Other**		

40 μm Multiscreen® 96 well mesh filter plate	Merck Millipore	Cat#MANMN4010
5 mL round bottom polystyrene tube with cell strainer snap cap	Corning	Cat#352003
125 mL Erlenmeyer cell culture flasks	Corning	Cat#CLS431143-1EA
High Throughput Sampler	BD Biosciences	Cat#339667
LSR Fortessa	BD Biosciences	RRID: SCR_018655
Micro test plate, 96-well, V-bottom	Sarstedt	Cat#82.1583
Polystyrene square 24-deepwell microplate	EnzyScreen	Cat#CR1421cl
Sandwich cover 24-deepwell microplate	EnzyScreen	Cat#CR1224
Universal clamp 24-deepwell microplate	EnzyScreen	Cat#CR1801h
Vacuum Hand Operator	Integra	Cat#155500

## Materials and equipment

**Table:** HEK293-6E cell culture media

Reagent	Amount
FreeStyle™ 293 Expression medium	1000 mL
Penicillin-Streptomycin (5.000 U/ml)	2 mL (final concentration 10 U/mL)

***Note:*** Store at 4°C for up to 1 month.

**Table:** PEI stock solution

Reagent	Amount
Branched Polyethylenimine (PEI), 25 kDa	0.45 g
Ultra-pure water	100 mL

***Note:*** Sterile-filter the solution using a 0.45 μm membrane filter before use. Store at 4°C up to 2 years.

**Table:** PEI working solution

Reagent	Amount
PEI stock solution	10 mL
Ultra-pure water	90 mL

***Note:*** Store at 20–25°C up to 8 weeks.

**Table:** Transfection mix

Reagent	Amount
HA-encoding plasmid	1 μg
DPBS	50 μL
PEI working solution	3.6 μL

***Note:*** Composition is given per 1 mL of cells. Adjust amounts accordingly when using different cell volumes. Prepare freshly and do not store long-term.

**Table:** FACS buffer

Reagent	Amount
PBS	500 mL
FBS	10 mL
EDTA 0.5 M	2 mL

***Note:*** Store at 4°C for up to 1 month.

## Step-by-step method details

### Transfection of HEK293-6E cells for surface protein expression

**Timing: 2 days**

This section describes the transfection of HEK293-6E suspension cells with individual antigen–fluorescent protein plasmids. Scale the volume of each transfection to the number of sera/antibodies to be tested.

***Note:*** The binding assay uses 60 μL per well at 1 × 10^6^ cells/mL for each antigen–fluorescent protein combination (see “Setting up the cell-based binding assay”). As HEK293-6E cells expand during the 48 h expression period, it is sufficient to plan the transfection with 50 μL per assay well at 0.8 × 10^6^ cells/mL (≈5 mL per 96-well plate, including controls).

***Note:*** We recommend 24-deepwell plates (2.5 mL per well) to streamline parallel handling. If larger cell volumes are required, transfections can be performed in 125 mL Erlenmeyer cell culture flasks.

1. Calculate the total amount/volume of cells needed for each antigen-fluorescent protein combination to be transfected.

**CRITICAL:** For the compensation controls, include one additional transfection of 2.5 mL HEK293-6E cells with a plasmid encoding for HA only without any fluorescent protein (HA-only cells, Steps 23–26).

2. Adjust the required volume of HEK293-6E cells to 0.8 × 10^6^ cells/mL in fresh culture medium and equilibrate for 1 h at 37°C, 6% CO_2_, 110 rpm shaking before transfection.
3. Transfect HEK293-6E cells with plasmids for surface protein expression.
  **CRITICAL:** Transfect cells in separate batches, using only one HA-encoding plasmid per batch. Do not co-transfect multiple plasmids into the same cells, as mixed HA expression will generate ambiguous populations and prevent reliable demultiplexing during flow cytometry.
  ***Note:*** Prepare the transfection mix using the transfection mix recipe provided in the materials and equipment section, and scale the volumes to the total culture volume for each HA-encoding plasmid.
  a. For each transfection, add the appropriate volume of DPBS to a separate reaction tube
  b. Add the required amount of plasmid DNA and mix by pipetting.
  c. Add PEI working solution to the tube and mix for 30 s by vortexing.
  d. Incubate for 10 min at 20–25°C.
  e. Add the transfection mixture dropwise to the cells while gently swirling.
  f. Incubate for 48 h at 37°C, 6% CO_2_ with shaking at 250 rpm shaking for 24-deepwell plates or 110 rpm for vented Erlenmeyer flasks.
    ***Optional:*** To estimate, whether transfections were successful, a small aliquot of cells may be examined by fluorescence microscopy 24 h after transfection (see Figure 2 for a representative fluorescence microscopy image). This step can be performed using any standard fluorescence microscope.
    **Figure 2:**
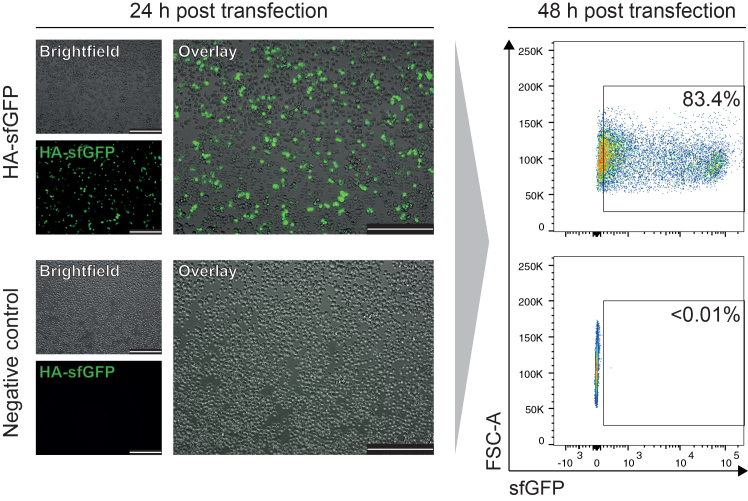
Representative fluorescence microscopy image of a successful HA-sfGFP transfection Cells were imaged 24 h after transfection with HA-sfGFP and compared with untransfected cells as a negative control. Brightfield, fluorescence, and overlay images are shown. In the corresponding experiment, the transfected cells yielded 83.4% sfGFP-positive cells by flow cytometry after 48 h, whereas the negative control contained <0.01% sfGFP-positive cells. Scale bars indicate 320.4 μm. Images were acquired on a Leica DMI 6000 B microscope using a Leica DFC365 FX camera and a 10× objective.

### Setting up the cell-based binding assay

**Timing: 6 h 30 min**

This section describes the multiplexing of transfected cells, the co-incubation with sera/plasma or monoclonal antibodies, and the staining of bound IgG using a fluorescently labeled anti-human IgG detection antibody.

**Figure 3:**
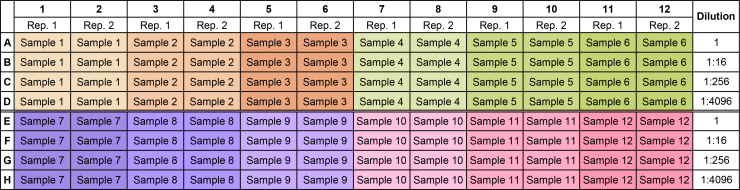
Example 96-well plate layout for serial titrations This layout allows testing of 12 individual samples in technical duplicates (Rep. 1/2) on a single 96-well plate (two blocks of six samples). Serum/plasma samples should be pre-diluted to 1:20 in PBS, and monoclonal antibodies should be pre-adjusted to a starting concentration of 25 μg/mL in PBS before preparing the dilution series. The example shown uses four dilution points generated by 1:16 serial dilutions (rows A–D and E–H); the layout and number of dilution points/replicates can be adapted to experimental needs and plate capacity.

***Note:***Figure 3 shows an example 96-well plate layout for testing twelve sera/plasma or monoclonal antibody samples across four dilutions on the same three antigen–fluorescent protein combinations in each well. The layout can be flexibly adapted (e.g., number dilutions, replicates) to match experimental needs and plate capacity.

***Note:*** We recommend technical duplicates to improve reproducibility and support robust curve fitting; however, duplicates may be omitted to increase throughput.

***Note:*** We recommend processing no more than eight 96-well plates in parallel.

***Note:*** Reconstitute Zombie NIR™ (hereafter ‘live/dead marker’) according to the manufacturer’s instructions in advance. Thaw the reconstituted reagent immediately before use.

***Note:*** If adapting this protocol to a different viability dye, use a live/dead marker compatible with fixation.

### Preparation of the samples

4. Harvest transfected HEK293-6E cells.
  a. Gently resuspend each transfected culture and transfer the cell suspension to an appropriate centrifugation vessel (e.g., a conical tube).
  b. Add 2 volumes of FACS buffer (relative to the transferred culture volume) to dilute the medium and mix gently.
  c. Centrifuge at 400 × *g* for 15 min at 4°C.
  d. Carefully decant the supernatant and resuspend the cell pellet in 2 mL FACS buffer per 5 mL of the initial culture volume.
  e. Count cells and adjust to 1 × 10^6^ cells/mL in FACS buffer.
  f. According to the planned plate layout, dispense 60 μL of each fluorescent protein–expressing cell population into the designated wells of a 96-well V-bottom plate (i.e., 3 × 60 μL per well to multiplex three antigen–fluorescent protein combinations)
  g. Store plates at 4°C, protected from light, until further processing.
5. Prepare serial dilutions of sera/plasma or monoclonal antibody samples in a 96-well V-bottom plate (example layout in Figure 3).
  ***Note:*** Include a known HA-binding monoclonal antibody, such as CR6261,^2^ as a positive control in each experiment to verify correct antigen expression and staining performance.
  ***Note:*** The starting concentrations and dilution factors are recommended values that worked robustly for HA detection and can be adjusted, for example, based on sample availability or expected binding strength. Prepare a final volume of ≥100 μL (e.g., 130 μL) per well to enable direct transfer to the assay plate (Step 8).
  a. Prepare 260 μL of each sample (for technical duplicates; ∼130 μL per well) in PBS with a starting dilution of 1:20 (sera/plasma) or a starting concentration of 25 μg/mL (monoclonal antibodies).
    i. Add 121.9 μL PBS to the designated wells in rows B–D and F–H.
    ii. Add 130 μL of each prepared sample (at the starting dilution/concentration) to the designated wells in rows A and E.
    iii. Perform a 1:16 serial dilution by transferring 8.1 μL from row A → B (and E → F), mix thoroughly by pipetting up and down 5 times and repeat the serial transfer B → C → D (and F → G → H), mixing after each step.
6. Centrifuge mixed cells in 96-well plates at 400 × *g* for 5 min at 4°C and aspirate the supernatant.

***Note:*** We recommend using an 8-channel vacuum manifold connected to a vacuum pump or, alternatively, a multichannel pipette to ensure consistent aspiration across wells.

**CRITICAL:** During all aspiration steps, do not disturb the cell pellet. Disturbing the pellet will cause cell loss and increased well-to-well variability (see Troubleshooting, Problem 2).

7. Transfer 100 μL of the prepared serial dilutions (Step 5) to the wells containing the cell pellets and resuspend the cells by pipetting.
8. Incubate for 30 min at 4°C, protected from light (in the fridge).
9. Wash by adding 190 μL FACS buffer to each well, centrifuge at 400 × *g* for 5 min at 4°C, and aspirate the supernatant.

**CRITICAL:** Thorough washing after incubation with sera/plasma or monoclonal antibodies is essential to minimize background and ensure accurate binding of the detection antibody (anti-human IgG, BV711; see Troubleshooting, Problem 4).

10. Repeat Step 9 using 290 μL FACS buffer.
11. Resuspend cells with 100 μL BD OptiBuild™ BV711 Mouse Anti-Human IgG (hereafter ‘detection antibody‘) diluted 1:300 in FACS buffer and mix by pipetting up and down 5 times.
12. Incubate for 30 min at 4°C, protected from light.
13. Repeat Step 9.
14. Resuspend cells in 100 μL live/dead marker diluted 1:600 in PBS, and mix by pipetting up and down 5 times.
15. Incubate for 30 min at 4°C, protected from light.
16. Repeat Step 9.
17. Resuspend cells in 100 μL BD Fixation buffer.

**CRITICAL:** BD Fixation buffer contains 4% PFA. PFA is hazardous and should be handled using appropriate personal protective equipment and disposed according to local waste regulations.

18. Incubate for 30 min at 4°C, protected from light.
19. Repeat Step 9.
20. Repeat Step 9 using 290 μL FACS buffer.
21. Resuspend cells in 120 μL FACS buffer.
22. Seal plates tightly and store at 4°C, protected from light, until flow cytometry acquisition.

### Preparation of gating and compensation controls

***Note:*** Compensation controls can be prepared in parallel with one another and the sample staining in the 96-well plates. However, for clarity, we describe the compensation control preparation as a separate step.

***Note:*** We recommend preparing fresh compensation controls for each experiment.

***Note:*** HA-only cells (no fused fluorescent protein) are used for the unstained control and live/dead single-stain control (Steps 23 and 26). In addition, HA-only cells are spiked into the fluorescent protein and detection antibody (anti-human IgG, BV711) single-stain controls (Steps 24 and 25) as an internal negative population.

**CRITICAL:** For gate setting, include a live/dead-only control for each fluorescent protein (Step 27).

23. Unstained control: Add 600 μL HA-only cells to a FACS tube.
  a. Store cells at 4°C, protected from light, until fixation (Step 28).
24. Fluorescent protein single-stain controls: For each antigen-fluorescent protein combination, add 400 μL fluorescent protein expressing cells plus 200 μL HA-only cells to a FACS tube.
  a. Store cells at 4°C, protected from light, until fixation (Step 28).
25. Detection antibody single-stain control: Add 400 μL of HA-only cells to a FACS tube.
  a. Centrifuge at 400 × *g* for 5 min at 4°C and carefully decant the supernatant.
  b. Add 400 μL of an HA-binding monoclonal antibody (100 μg/mL), resuspend, and incubate for 30 min at 4°C.
  c. Wash twice with 2 mL FACS buffer (centrifuge at 400 × *g* for 5 min at 4°C and carefully decant the supernatant after each wash).
  d. Resuspend cells in 400 μL of the detection antibody 1:300 diluted with FACS buffer and incubate for 30 min at 4°C.
  e. Wash once as in Step 25c.
  f. Add 200 μL HA-only cells plus 300 μL FACS buffer and resuspend cells by pipetting up and down 5 times.
  g. Store cells at 4°C, protected from light, until fixation (Step 28).
26. Live/dead single-stain control: Add 600 μL HA-only cells to a FACS tube.
  a. Centrifuge at 400 × *g* for 5 min at 4°C and carefully decant the supernatant.
  b. Add 600 μL live/dead marker diluted 1:600 in PBS, resuspend, and incubate for 30 min at 4°C.
  c. Wash with 2 mL of FACS buffer, centrifuge at 400 × *g* for 5 min at 4°C, and carefully decant the supernatant.
  d. Resuspend cells in 300 μL FACS buffer
  e. Store cells at 4°C, protected from light, until fixation (Step 28).
27. Fluorescent protein gating controls (live/dead-only): For each antigen-fluorescent protein combination, add 200 μL fluorescent protein–expressing cells to separate FACS tubes.
  a. Centrifuge at 400 × *g* for 5 min at 4°C and carefully decant the supernatant.
  b. Add 200 μL live/dead marker diluted 1:600 in PBS, resuspend, and incubate for 30 min at 4°C.
  c. Wash with 2 mL of FACS buffer, centrifuge at 400 × *g* for 5 min at 4°C and carefully decant the supernatant.
  d. Resuspend cells in 300 μL FACS buffer.
  e. Store cells at 4°C, protected from light, until fixation (Step 28).
28. Fix cells of all gating and compensation controls (Steps 23-27).
  **CRITICAL:** BD fixation buffer contains 4% PFA. Handle with appropriate personal protective equipment and dispose of waste according to local regulations.
  a. Centrifuge at 400 × *g* for 5 min at 4°C and carefully decant the supernatant.
  b. Resuspend cells in 400 μL BD fixation buffer.
  c. Incubate for 30 min at 4°C, protected from light.
29. Wash twice with 2 mL FACS buffer (centrifuge at 400 × *g* for 5 min at 4°C and carefully decant the supernatant after each wash).
30. Resuspend cells in 250 μL FACS buffer, filter into FACS tubes, and store at 4°C, protected from light, until flow cytometry acquisition.

**Pause point:** Plates and gating/compensation controls can be stored at 4°C, protected from light, for up to 1 week. However, we recommend acquisition within 2 days for optimal signal stability.

### Flow cytometry analysis of samples

**Timing: 45 min per plate**

This section describes the flow cytometric acquisition on a BD LSRFortessa equipped with a BD High Throughput Sampler (HTS) and configuration as in Table 1.

***Note:*** When using alternative cytometers, fluorophore assignments should be adjusted according to the available laser and filter configuration to minimize spectral overlap and maintain clear separation of fluorescent protein signals and the detection antibody.

31. Set up the flow cytometer for analysis according to the manufacturer’s instructions.
32. Immediately before acquisition, pass samples through a filter to remove aggregates.
  a. For compensation controls, we recommend FACS tubes with a 35 μm cell-strainer cap.
  b. For 96-well plates, we recommend 40 μm filter plates.
  c. After filtering, pulse spin all 96-well plates at 400 x g and transfer samples to a fresh 96-well V-bottom plate for acquisition.
33. Use the compensation controls (Steps 23–26) to adjust detector voltages and calculate a new compensation matrix.

***Note:*** The three fluorescent proteins used in this assay exhibit high signal intensities. Therefore, lower detector voltages are typically required to keep signals within the linear detection range and avoid saturation.

34. Acquire the fluorescent protein gating controls stained with live/dead marker (Zombie NIR, Step 27).
35. Set the gates as shown in Figure 4:
  a. FSC-A vs. SSC-A: Gate on the main cell population and exclude debris.
  b. FSC-A vs. FSC-H: Exclude doublets.
  c. FSC-A vs. APC-Cy7: Gate on Zombie NIR–negative cells (Zombie NIR is detected in the APC-Cy7 channel; intact cells show low APC-Cy7 signal).
  d. Gate on HA1-mTagBFP2/anti-IgG-positive cells:
    i. FSC-A vs. BV421: Gate on mTagBFP2-positive cells.
      ***Note:*** Set this gate using live/dead–stained controls that are negative for mTagBFP2 (sfGFP-only or dTomato-only controls, Step 27).
    ii. BV421 vs. FITC: Exclude spillover-derived false positives from sfGFP.
    iii. BV421 vs. PE: Exclude spillover-derived false positives from dTomato.
    iv. BV421 vs. BV711: Quantify IgG binding as BV711-positive events within the mTagBFP2-positive gate.
      ***Note:*** Define the BV711-positive gate using the corresponding negative control (mTagBFP2-positive cells, live/dead–only; Step 27) and set the threshold so that 0.1%–0.5% of events from this negative control fall within the BV711-positive gate.
  e. Gate on HA2-sfGFP/anti-IgG-positive cells:
    i. FSC-A vs. FITC: Gate on sfGFP-positive cells.
      ***Note:*** Set this gate using live/dead–stained controls that are negative for sfGFP (mTagBFP2-only or dTomato-only controls, Step 27).
    ii. FITC vs. BV421: Exclude spillover-derived false positives from mTagBFP2.
    iii. FITC vs. PE: Exclude spillover-derived false positives from dTomato.
    iv. FITC vs. BV711: Quantify IgG binding as BV711-positive events within the sfGFP-positive gate.
      ***Note:*** Define the BV711-positive gate using the corresponding negative control (sfGFP-positive cells, live/dead–only; Step 27) and set the threshold so that 0.1%–0.5% of events from this negative control fall within the BV711-positive gate.
  f. Gate on HA3-dTomato/anti-IgG-positive cells:
    i. FSC-A vs. PE: Gate on dTomato-positive cells.
      ***Note:*** Set this gate using live/dead–stained controls that are negative for dTomato (mTagBFP2-only or sfGFP-only controls, Step 27).
    ii. PE vs. BV421: Exclude spillover-derived false positives from mTagBFP2.
    iii. PE vs. FITC: Exclude spillover-derived false positives from sfGFP.
    iv. PE vs. BV711: Quantify IgG binding as BV711-positive events within the dTomato-positive gate.
      ***Note:*** Define the BV711-positive gate using the corresponding negative control (dTomato-positive cells, live/dead–only; Step 27) and set the threshold so that 0.1%–0.5% of events from this negative control fall within the BV711-positive gate.

**Figure 4:**
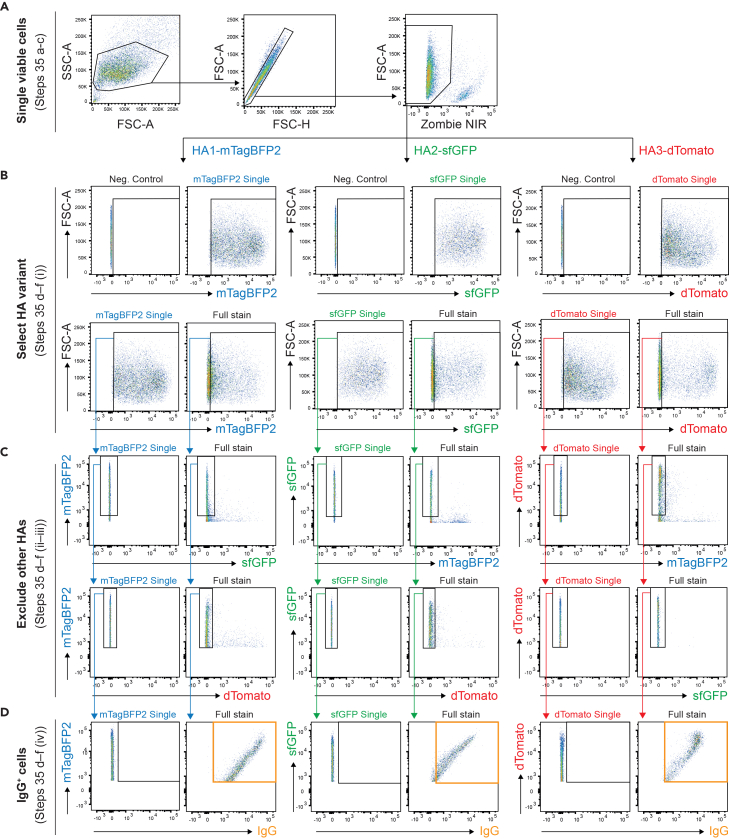
Representative gating strategy for the cell-based binding assay Gating was performed sequentially as follows. (A) The main HEK293-6E cell population was identified by FSC-A/SSC-A, doublets were excluded by FSC-H/FSC-A, and viable cells were gated based on Zombie NIR (APC-Cy7 channel). (B) To define gates for individual fluorescent protein–expressing populations (mTagBFP2, sfGFP, or dTomato), single-color controls were used. For each HA variant, a non-multiplexed sample expressing one of the other fluorescent proteins and stained with live/dead served as a negative control to discriminate transfected from untransfected cells. This should result in nearly all cells of the respective fluorescent protein gating control (live/dead-only, Step 27) falling into the gate (top right and bottom left panel for each fluorescent protein). Bottom right panels for each fluorescent protein show representative gates of a fully stained sample. (C) Subsequently, dimly expressing cells and spillover-derived false-positive signals from the remaining fluorescent proteins were excluded to obtain clean single-positive populations. Representative gates are shown for the corresponding fluorescent protein gating (live/dead-only) control and a fully stained sample. (D) IgG-binding cells were identified within each fluorescent protein–positive population by BV711 (anti-human IgG) gating; the BV711-positive threshold was defined using the corresponding fluorescent protein–positive live/dead-only control (Step 27). Representative gates are shown for the corresponding fluorescent protein gating (live/dead-only) control and a fully stained sample. Adapted with modifications from Daniel et al., 2026.^1^
36. Acquire the plate. Acquisition parameters may need to be optimized depending on the instrument and HTS configuration. For the BD LSRFortessa with HTS, we used:
  a. Throughput Mode set to standard.
  b. Sample flow rate set to 3.
  c. Sample volume (μL) set to 60.
  d. Mixing volume (μL) set to 50.
  e. Mixing speed (μL/sec) set to 180.
  f. Number of mixes set to 3.
  g. Wash volume (μL) set to 400.
  h. Enable BLR set to on.
  i. BLR period set to 50.
37. Set the stopping gate to record 10,000 viable cells per well (after viability gating, Step 35c) to ensure sufficient events for downstream analysis.

**Table 1:** BD LSRFortessa instrument configuration

Channel name	Laser	Filter	Fluorophore/Fluorescent protein
BV421	405 nm	450/50	mTagBFP2
BV711	405 nm	710/50	Anti-IgG
FITC	488 nm	530/30	sfGFP
PE	561 nm	586/15	dTomato
APC-Cy7	640 nm	780/60	Zombie NIR

## Expected outcomes

Successful execution of this protocol results in robust and reproducible detection of antigen-specific IgG binding to viral surface proteins expressed on HEK293-6E cells. A key determinant of assay performance is transfection efficiency, as a high fraction of fluorescent reporter–positive cells ensures sufficient surface expression of the viral target antigen. Under optimal conditions, transfection efficiencies typically exceed 90%, although efficiencies of ≥60% are sufficient to generate reliable binding signals. Lower transfection efficiencies reduce the proportion of antigen-expressing cells and may require acquisition of a higher total number of events during flow cytometric analysis. Surface expression of the antigen constructs may also be verified prior to experiments, for example by staining transfected cells with a known HA-binding antibody and analyzing surface accessibility by flow cytometry.

After seeding and centrifugation in 96-well plates, clearly visible and compact cell pellets should be observed. When acquisition settings and cell numbers described in this protocol are followed, sufficient events are typically recorded to allow reliable gating of at least 10,000 viable cells per condition. Antigen-reactive samples yield a clear shift in BV711 signal within the respective fluorescent protein–positive population, whereas negative control samples remain at baseline. In reactive samples, BV711 signal intensity commonly correlates with fluorescent reporter intensity, reflecting increased antibody binding on cells expressing higher levels of surface antigen.

**Figure 5:**
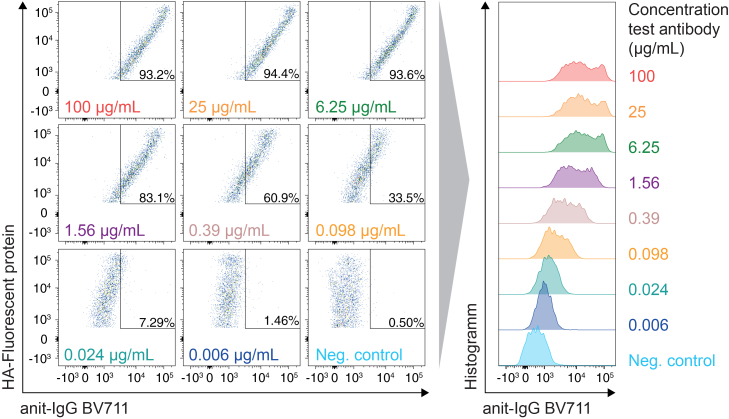
Representative flow cytometry plots demonstrating dilution-dependent reduction of antigen-specific binding Representative flow cytometry plots of a serial dilution of the HA-reactive antibody CR6261^2^ tested against HA A/Hawaii/70/2019 (H1N1)-mTagBFP2. After sequential gating on viable, single HEK293-6E cells and fluorescent protein–positive populations, BV711 signal (anti-human IgG) was analyzed within the antigen-expressing cells. With decreasing CR6261 concentration, a progressive reduction in BV711-positive events is observed, resulting in a stepwise decrease in signal intensity across the dilution series. These dilution-dependent shifts are also visible in the BV711 histograms (right panel) and form the basis for quantitative analysis and subsequent curve fitting (see quantification and statistical analysis)

Figure 5 shows a representative eight-point dilution series of the HA-reactive monoclonal antibody CR6261^2^ tested against HA A/Hawaii/70/2019 (H1N1)–mTagBFP2, illustrating the dilution-dependent reduction in BV711-positive events. The resulting quantitative values follow sigmoidal binding curves that allow determination of half maximal effective concentration (EC_50_) or dilution (ED_50_) values (see quantification and statistical analysis). Under optimized conditions, technical replicates typically vary by ≤5%. Larger deviations may indicate pipetting inaccuracies, sample heterogeneity, suboptimal washing, or reduced transfection efficiency.

## Quantification and statistical analysis

Flow cytometry data are analyzed using the sequential gating strategy described in Figure 4. Viable, single HEK293-6E cells are identified and separated into fluorescent protein–positive populations. IgG binding is quantified within each fluorescent protein–positive gate using the BV711 signal of the anti-human IgG detection antibody.

Binding can be reported either as the percentage of BV711-positive events or as BV711 median fluorescence intensity (MFI). Percentage-based readouts are typically robust, whereas MFI may provide additional quantitative resolution in some settings. Background staining is ideally assessed using wells stained with the live/dead marker (Zombie NIR) and the detection antibody (anti-human IgG, BV711), but without serum/plasma/monoclonal antibody (secondary-only control). In our case, secondary-only controls were indistinguishable from live/dead-only controls. Therefore, we use live/dead-only wells as the negative control. The BV711 positivity threshold is based on the negative control such that 0.1%–0.5% of events fell within the BV711-positive gate (Step 35). Given this minimal background, we do not subtract background from the raw data, as we found that it does not measurably affect downstream calculations.

To determine EC_50_ (monoclonal antibodies) or ED_50_ (sera/plasma) values, binding signals are plotted against antibody concentration (monoclonal antibodies) or reciprocal dilutions (sera/plasma). Nonlinear regression using a four-parameter logistic (4PL) model is applied to derive EC_50_/ED_50_ values. Data should be excluded from EC_50_/ED_50_ analysis if no clear dynamic range is observed, if signal does not exceed background across the dilution series, or if replicate variability is high. Technical replicates should be analyzed independently and averaged prior to reporting. Consistent dynamic range and sigmoidal curve shape indicate reliable assay performance.

**Figure 6:**
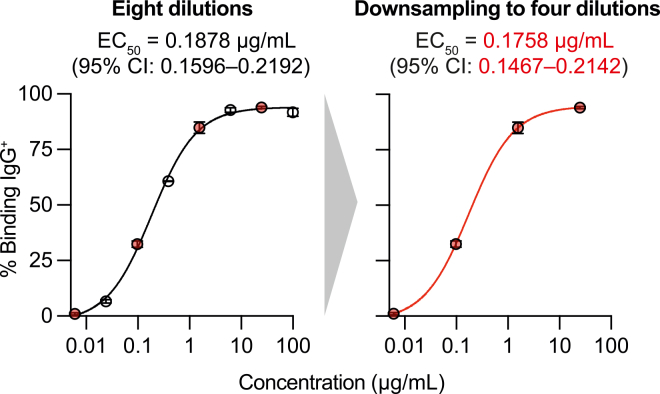
Robust EC_50_ estimation from percentage-based IgG-positive gating Titration curves for the HA-reactive monoclonal antibody CR6261 were generated by plotting the percentage of BV711-positive cells within the fluorescent protein–positive population (% Binding IgG^+^) versus antibody concentration, using the same gated data shown in Figure 5. Data points represent mean values of two biological replicates (*n* = 2), and error bars indicate standard deviations (SD). Curves were fitted with a four-parameter logistic (4PL) model to determine EC_50_ values (95% CI). The full eight-point titration is shown on the left; the same dataset downsampled to four dilution points is shown on the right, yielding a comparable EC_50_ estimate (selected points are highlighted in red).

A representative curve fit derived from the gated data of CR6261 shown in Figure 5 is illustrated in Figure 6. The downsampling of the corresponding eight-point dilution series to four dilution points shows that four-point titrations are sufficient for robust EC_50_ estimation in this assay. Similar results were obtained for polyclonal sera or plasma, yielding comparable ED_50_ estimates. Therefore, we typically use (and recommend) four dilution points, as illustrated in the example layout (Figure 3), to increase overall throughput.

## Limitations

This protocol was optimized for the analysis of IgG binding to influenza A virus hemagglutinin (HA) expressed on the surface of HEK293-6E suspension cells. While the modular plasmid design allows straightforward exchange of the viral target antigen, assay performance may vary depending on the biochemical properties of the substituted surface protein. In particular, some viral glycoproteins may require stabilizing mutations, optimized secretion signals, or modifications of transmembrane and cytoplasmic domains to ensure efficient surface expression and preservation of relevant epitopes. Therefore, when switching to alternative viral proteins, additional optimization and validation of surface expression and antibody accessibility may be required.

A further limitation is the reliance on suspension culture and shaking incubation conditions for HEK293-6E cells. Laboratories without access to suspension culture infrastructure may need to adapt the protocol to adherent cell lines. However, such adaptations can affect assay robustness and throughput. High-throughput acquisition may be less reliable with adherent cells due to increased aggregation, adherence to plastic surfaces, or reduced viability during repeated centrifugation and resuspension steps. In these cases, additional optimization of handling and acquisition settings is required.

Finally, quantitative readouts depend on instrument configuration and analysis parameters. Detector voltages, compensation settings, gating thresholds, and background staining directly influence the percentage of BV711-positive events and fluorescence intensity values. Absolute values may therefore differ between instruments or laboratories if acquisition and gating strategies are not standardized. Consistent instrument settings, inclusion of appropriate controls, and uniform gating across experiments are required to ensure reproducible and comparable results.

## Troubleshooting

### Problem 1

Low transfection efficiency of HEK293-6E cells (Step 3).

### Potential solution

Transfection efficiency strongly depends on cell density and overall cell health. Cells that exceeded 1.8 × 10^6^ cells/mL prior to transfection frequently show reduced transfection efficiency that may persist even after dilution. Ensure strict monitoring of cell density during routine culture and avoid overgrowth. If transfection efficiency drops below 60%, discard the culture and thaw a new vial. Additionally, verify plasmid DNA quality (purity and integrity) and confirm proper preparation and storage of PEI working solution.

### Problem 2

Low number of viable cells during acquisition; fewer than 10,000 viable fluorescent protein–positive cells are recorded (Step 37).

### Potential solution

**Figure 7:**
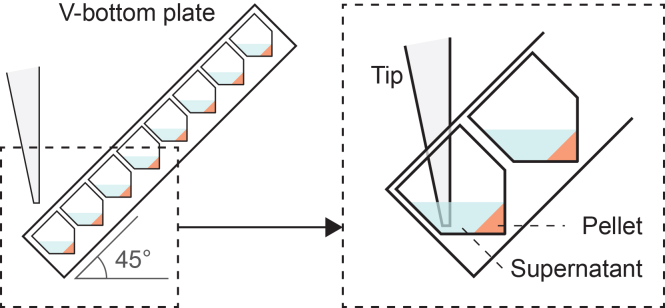
Aspiration of supernatant without disturbing cell pellets The 96-well plate is held at a 45° angle, and the supernatant is aspirated from the corner of the V-bottom plate to minimize cell loss.
• Process plates in batches to minimize time between centrifugation and aspiration, as prolonged incubation in supernatant can destabilize the pellet. Confirm correct centrifugation settings (400 × *g*, 4°C). If event numbers remain insufficient, increase acquisition volume or reduce washing stringency.

• Cell loss most commonly occurs during aspiration steps. Avoid disturbing the pellet during supernatant removal. We recommend to hold the 96-well plate in a 45-degree angle and aspirate from the corner of the V-bottom plate (Figure 7).

### Problem 3

Irregular signal acquisition or unstable flow during measurement (Step 36).

### Potential solution

Air bubbles or cell aggregates may cause unstable flow and artificial signal fluctuations. Inspect FSC-A versus time plots for flow stability. Filter samples immediately before acquisition and avoid introducing bubbles during pipetting. If artifacts are detected, exclude affected time intervals during analysis.

### Problem 4

High background or paradoxically higher IgG binding at higher dilutions (Steps 7-10).

### Potential solution

Insufficient washing after incubation with sera, plasma, or monoclonal antibodies may leave residual IgG in solution. Free IgG can bind and saturate detection antibody, reducing measurable signal in low-dilution samples. Implement rigorous washing with complete supernatant removal. Increase wash volume or number of washing steps if required. Verify correct preparation and dilution of the detection antibody.

### Problem 5

No detectable IgG signal in any sample (Step 36).

### Potential solution

Include positive and negative controls in every experiment to verify correct staining and acquisition. Confirm correct dilution and incubation of detection antibody and live/dead marker. Check detector voltages and compensation settings, as excessively low voltages may suppress BV711 signal. Ensure correct identification of fluorescent protein–positive populations during gating (Step 33).

### Problem 6

Poor compensation or unexpected fluorescence spillover (Steps 23-27, 33, and 35).

### Potential solution

Compensation requires high-quality single-stain controls containing clearly positive and negative populations. Prepare fresh single-stain controls for each experiment. Verify correct detector assignment according to the instrument’s optical configuration. Adjust voltages prior to compensation to ensure adequate signal separation.

### Problem 7

Inconsistent EC_50_/ED_50_ values or poor curve fitting during quantitative analysis (quantification and statistical analysis section).

### Potential solution

Unreliable curve fitting may result from insufficient dynamic range, inaccurate dilution series preparation, or high replicate variability. Ensure precise pipetting during serial dilutions (Step 5). Verify that the tested dilution range captures both maximal and minimal binding signals. Exclude outlier wells only if justified by technical error. Maintain consistent gating across dilution series to ensure comparability of calculated percentages.

## Resource availability

### Lead contact

Further information and requests for resources and reagents should be directed to and will be fulfilled by the lead contact, Christoph Kreer (christoph.kreer@uk-koeln.de).

### Technical contact

Technical questions on executing this protocol should be directed to and will be answered by the technical contact, Leon Ullrich (leon.ullrich@uk-koeln.de).

### Materials availability

Expression plasmids pHA1, pHA3, and pHA5 (pcDNA3.1-HA-H1N1-sfGFP, pcDNA3.1-HA-H3N2-mTagBFP2, and pcDNA3.1-HA-H5N1-dTomato) have been deposited at Addgene (accession nos. 253733–253735). Requests for any additional material will be fulfilled by the lead contact upon request but might be restricted by sample availability or data protection, and may require a material transfer agreement (MTA) for non-commercial usage.

### Data and code availability

This study did not generate new datasets or original custom code. The hemagglutinin sequences used in this protocol were originally derived from GISAID and are described in detail in Daniel et al.^1^ No new sequence data were generated as part of this work. Data supporting the original application of this assay are available in Daniel et al.^1^

## Acknowledgments

We thank Lisa Kottege, Hanna Janicki, Denise Engel, and Banu Meiners for excellent technical assistance. We thank Martin Hufbauer and Xenia Spiliopoulou for their technical assistance with fluorescence microscopy. This work was funded by the 10.13039/501100001659German Research Foundation (10.13039/501100001659DFG) under the CRC1310 (to C.K. and F.K.). Elements of the graphical abstract were created with Biorender.com. We gratefully acknowledge the authors and submitting laboratories of the sequences accessed via GISAID, as detailed in Daniel et al.^1^

## Author contributions

Conceptualization, L.U. and C.K.; methodology, L.U., K.D., A.-L.S., M.G., and C.K.; investigation, L.U., K.D., and C.K.; formal analysis, L.U. and C.K.; visualization, L.U. and C.K.; funding acquisition, F.K. and C.K.; project administration, C.K.; resources, F.K. and C.K.; supervision, C.K.; writing – original draft, L.U. and C.K.; writing – review and editing, all authors.

## Declaration of interests

C.K. and F.K. are listed as inventors on patents related to antiviral monoclonal antibodies and have received payments from the University of Cologne for licensed patents. F.K. is the founder and shareholder of Togontech, a company engaged in the development of antibodies for prevention and therapy. The authors declare that these activities are not directly related to the method described in this protocol.

## Declaration of generative AI and AI-assisted technologies in the writing process

During the preparation of this work, the authors used ChatGPT (OpenAI) to support English language editing and grammatical corrections. After using this tool, the authors reviewed and edited the content as needed and take full responsibility for the content of the published article.
